# Porcelain Inlays

**Published:** 1899-05

**Authors:** H. S. Guilford

**Affiliations:** Philadelphia.


ARTICLE III.

### PORCELAIN INLAYS.

H. S. GUILFORD, D. D. S., PH. D., PHILADELPHIA.

The latest methods of making porcelain inlays, and undoubtedly the best and most promising one yet devised, is that of Dr. N. S. Jenkins, of Dresden, Saxony. It resembles previous methods in that it consists in making a metal matrix of the cavity, and into this fusing a porcelain body of proper shade and consistency; but it differs considerably from any other in many of its details.

Dr. Jenkins was led to experiment in the making of inlays by the desire of his patients to avoid the conspicuousness of gold, and by his failure to obtain satisfactory results by the methods previously employed. His experiments extended through several years, but in the end his efforts were drowned with success.

In making the matrix he used gold foil No. 30, having found that platinum lacked the same degree of adaptability that gold possessed, and he also found that gold heavier than No. 30 did not yield as good results.

In the matter of porcelain body he noticed that lowfusing bodies previously used were lacking in life-like color snd strength. Through experiment, and by consultation with those familiar with the making of porcelain ware, he at last hit upon a combination of ingredients which gave him the desired texture and appearance, and also fused at a considerable lower heat than gold. In addition to this his combination was subject to less shrinkage in cooling than any bodies previously used.

These combined improvements enabled him to prepare an inlay of any desired shade, and one that would so closely fit the cavity as to make it almost indistinguishable from the tooth in which it is placed.

For the fusing of the porcelain body, Dr. Jenkins has devised an oven, heated by a jet of gas and air.

The furnace is shaped like a muffle, about three by three by five inches, is made of Russia iron, and lined with asbestos cloth. The jet of gas and air is supplied by means of an ordinary gas blow-pipe, such as is used in the laboratory for soldering, and the air pressure is obtained from a foot-bellows. The furnace is supported on feet, and in its base is a hole, of an inch or less in diameter, through which the jet from the blow-pipe enters.

In making the gold matrix, the piece of foil is pressed to the shape of the cavity with cotton or spunk, held in a pair of blunt tweezers, and the margins of the cavity sharply outlined in the gold by burnishing it over the edges.

For holding the matrix during the baking, a platinum spoon is used, having a long handle and a hemispherical bowl about seven-eighths of an inch in diameter. In this is placed fine asbestos, mixed with water, and the matrix gently imbedded in it. Heating over a gas flame dries out the moisture, and the matrix is then ready for the porcelain.

Instead of mixing his body with water, as has heretofore been customary, Dr. Jenkins uses alcohol, on account of its more ready evaporation. Moistening the porcelain powder with alcohol on a glass slab, it is placed in the matrix and gently dried over a flame. The platinum spoon, with its matrix and body, is now inserted in the open mouth of the muffle and the flame applied by the blow-pipe. In a few minutes the porcelain is fused and allowed to cool. More porcelain is now added and the operation repeated.

In order to secure thorough fusing and make up for shrinkage, the porcelain is added and baked in two or three portions.

When completed, the foil matrix is stripped from the inlay, and the latter inserted into the cavity to see that all is satisfactory. Before placing the inlay permanently the cavity will have to be enlarged inwardly, or roughened with a burr, and the inlay may also require to have a groove cut into its under portion with a delicate corundum wheel. If it has not been done previously, the rubber-dam should next be applied and the cavity thoroughly dried. Zinc phosphate of a light color is now mixed to creamy consistency, some of it applied to the margins of the cavity, and some to the under side of the inlay, and the latter quickly pressed into position. Pressure upon the inlay, to force it home and expel any excess of cement, can best be exerted with a piece of orange-wood.

After the cement has hardened and any surplus removed from the margins, the case is complete.

Dr. Jenkins has prepared his porcelain in a dozen or two different shades, so that, with these, or a combination of any two of them, any desired shade can be obtained. A large amount of skill and experience is necessary in order to obtain just the shade desired, and much patience is required to secure a thoroughly correct matrix. The foil must not move while it is being adapted to the cavity, and in removing it great care needs to be exercised not to change its form. So, also, in placing and imbedding the matrix in the asbestos in the platinum spoon, delicate manipulations is absolutely necessary.

Again, practice alone can impart the skill and good judgment necessary in placing the proper amount of body in the matrix, so that, when fused, the inlay will entirely fill, but not more than fill, the matrix. If two much body be placed in the matrix, the inlay will overlap the margins and prove a failure; whereas, if too little is used, the inlay will not entirely fill the cavity.

Dr. Jenkins thinks that his furnace should, in all cases, be used, in order to secure satisfactory results; but there seems to be no good reason why a Downie or Land furnace, or a Custer electric oven should not be employed with equally good results. The Jenkins furnace may be used anywhere that a gas supply may be obtained, and this fact will make it more generally useful to practitioners who cannot obtain the electric current. It also does its work very quickly, for with it the porcelain body can be fused in a few minutes. Fusing in an electric oven will take longer, which would prove an objection where it is desired to take and set the inlay at one sitting.

The zinc cement with which the inlay is secured in the cavity is, of course, perishable; but where there is a close adaptation of the inlay to the cavity, the line of cement exposed will be very fine, and, if dissolved out in time, can easily be replaced without removing the inlay.

Inlay work is most called for in the interior teeth, especially upon their labial and approximal surfaces. Farther back in the mouth it would possess no special advantage over the metals now generally employed. For the restoration of corners of teeth an inlay can hardly be made strong enough in its anchorage to resist the strain of mastication, unless the tooth be pulpless so that the cavity can be greatly enlarged inwardly.

The making and setting of an inlay will generally consume more time than the insertion of a gold filling, but experience will bring with it rapidity of manipulation as in all other processes.—*The Stomatologist.*

